# Challenges and potential solutions for physician suicide risk factors in the COVID-19 era: psychiatric comorbidities, judicialization of medicine, and burnout

**DOI:** 10.47626/2237-6089-2021-0293

**Published:** 2023-05-02

**Authors:** Dante Duarte, Mirret M. El-Hagrassy, Tiago Couto, Wagner Gurgel, Luciano Minuzzi, Karen Saperson, Humberto Corrêa

**Affiliations:** 1 Spaulding Hospital Harvard Medical School Boston MA USA Spaulding Hospital, Harvard Medical School, Boston, MA, USA.; 2 Department of Psychiatry and Behavioural Neurosciences McMaster University Hamilton ON Canada Department of Psychiatry and Behavioural Neurosciences, McMaster University, Hamilton, ON, Canada.; 3 Neurology Department University of Massachusetts Medical School Worcester MA USA Neurology Department, University of Massachusetts Medical School, Worcester, MA, USA.; 4 Universidade Federal de Uberlândia Uberlândia MG Brazil Universidade Federal de Uberlândia, Uberlândia, MG, Brazil.; 5 Universidade de São Paulo São Paulo SP Brazil Universidade de São Paulo, São Paulo, SP, Brazil.; 6 Mood Disorders Program and Women’s Health Concerns Clinic, St. Joseph’s Healthcare Hamilton ON Canada Mood Disorders Program and Women’s Health Concerns Clinic, St. Joseph’s Healthcare Hamilton, ON, Canada.; 7 Faculdade de Medicina Universidade Federal de Minas Gerais Belo Horizonte MG Brazil Faculdade de Medicina, Universidade Federal de Minas Gerais, Belo Horizonte, MG, Brazil.

**Keywords:** Physician suicide, COVID-19, burnout, judicialization of medicine, healthcare organization, depression

## Abstract

**Introduction:**

Suicide among physicians constitutes a public health problem that deserves more consideration. A recently performed meta-analysis and systematic review evaluated suicide mortality in physicians by gender and investigated several related risk factors. It showed that the post-1980 suicide mortality was 46% higher in female physicians than among women in the general population, while the risk in male physicians was 33% lower than among men in general, despite an overall contraction in physician mortality rates in both genders.

**Methods:**

This narrative review was conducted by searching and analyzing articles/databases that were relevant to addressing questions raised by a prior meta-analysis and how they might be affected by COVID-19. This process included unstructured searches on Pubmed for physician suicide, burnout, judicialization of medicine, healthcare organizations, and COVID-19, and Google searches for relevant databases and medical society, expert, and media commentaries on these topics. We focus on three factors critical to addressing physician suicides: epidemiological data limitations, psychiatric comorbidities, and professional overload.

**Results:**

We found relevant articles on suicide reporting, physician mental health, the effects of healthcare judicialization, and organizational involvement on physician and patient health, and how COVID-19 may impact such factors. This review addresses information sources, underreporting/misreporting of physician suicide rates, inadequate diagnosis and management of psychiatric comorbidities and the chronic effects on physicians’ work capacity, and, finally, judicialization of medicine and organizational failures increasing physician burnout. We discuss these factors in general and in relation to the COVID-19 pandemic.

**Conclusions:**

We present an overview of the above factors, discuss possible solutions, and specifically address how COVID-19 may impact such factors.

## Introduction

Suicide is among the leading causes of mortality worldwide^1^ and the US age-adjusted suicide rate in 2018 was the highest since 1941.^2^ Suicide among physicians constitutes a public health problem and modifiable risk factors play a clear moderating role. We recently performed a meta-analysis and systematic review evaluating suicide mortality in physicians by gender and investigating several relevant risk factors. Our results showed that the post-1980 suicide mortality was 46% higher in female physicians than among women in the general population, while the risk in male physicians was 33% lower than among men in the general population.^3^

Psychiatric comorbidities were one of the physician suicide risk factors identified in our systematic review^3^ and need further consideration, as do certain factors that were not addressed: judicialization of medicine, organizational oversight, and burnout. Moreover, the COVID-19 pandemic ravaging the world has had harmful effects on the mental health of physicians, especially frontline physicians.^4^ For instance, China was the first country hit by the pandemic and we found that rates of depression and anxiety symptoms were as high as 50% in 1,257 physicians and healthcare workers in China, while 70% were in distress.^5^ A similar trend was observed in Italy, which was one of the most affected countries in Europe, where nearly 50% of 1,379 healthcare workers reported posttraumatic stress symptoms and almost 30% reported symptoms of depression and anxiety.^6^

In this narrative review, we therefore searched and analyzed databases for relevant articles with the intention of addressing these risk factors, by investigating their main issues and potential solutions. We then presented these risk factors considering the current COVID-19 pandemic and how it may impact the likelihood of physician death by suicide.

This process included unstructured searches on Pubmed for physician suicide, judicialization of medicine, healthcare organization oversight, burnout, and COVID-19 and Google searches for relevant databases as well as medical society, expert, and media commentaries on these topics. We postulated that these factors may have been worsened by the impact of the COVID-19 pandemic on physician workforces and wellbeing.

## Risk factors

### Overview

#### Psychiatric comorbidities

Most patients who die by suicide suffer from psychiatric comorbidities.^7,8^ Physicians, in particular, often have unmanaged (undiagnosed or untreated) psychiatric disorders, primarily depression and substance abuse (alcohol and drugs).^9^ At early stages of their careers, medical practitioners already have higher levels of depression than the general population.^10,11^ A series of recent meta-analyses estimated the prevalence of depression at 27.2% in medical students and 28.8% in resident physicians, ranging from 9.3% to 55.9% and 20.9% to 43.2%, respectively, depending on the instrument used.^10,11^ Moreover, depression prevalence rates in resident physicians increased by calendar year (0.5% increase per year).^11^ If not properly treated, these psychiatric conditions become chronic, with potential for significant harm to physicians’ practices and reputations and to patient safety.^12^ Nevertheless, we could not find any meta-analyses investigating practicing physicians’ depression symptoms or disorders.

The American Foundation for Suicide Prevention (AFSP) published *Facts about Mental Health and Suicide Among Physicians*.^9^ One of the facts listed is that among people whose death was by suicide, physicians were less likely to have been receiving mental health treatment than non-physicians, despite similar rates of depression.^13^ The same problem was observed in medical students.^10^ Without effective long-term therapy for underlying psychiatric issues, physicians are more likely to self-medicate for depression, anxiety, insomnia, etc. This may temporarily alleviate symptoms but can have disastrous long-term consequences. Of 601 US anesthesiologists in a retrospective cohort study who developed substance use disorders, 114 (19%) died from substance use-related factors during follow-up.^14^ The prevalence of physician alcohol and drug abuse may relate to self-medicating symptoms of depression, anxiety, and insomnia, although attempted cognitive enhancement through psychostimulants is also common among medical students and physicians.^15,16^ A Swiss study found that regular use of hypnotics and sedatives (mainly benzodiazepines) was significantly higher among doctors than in the general population.^17^ Psychiatry is the specialty with the highest percentage of daily use of such medications.^11^ In random US samples of physicians and students, 10% of 337 physicians and 16% of 381 medical students reported psychoactive drug use once a month or more.^18^

Despite substantial psychiatric comorbidities, physicians tend not to seek care for their own health: nearly 40% of 5,829 surveyed physicians reported reluctance to seek formal mental healthcare due to “concerns about repercussions to their medical licensure”; the study found that medical licensure application questions regarding psychiatric conditions were a barrier to physicians seeking help.^19^

Meanwhile, people close to physicians who died of suicide were often unaware of their suffering, possibly due to physicians’ pretense of being well-adjusted.^20^ Physician personality factors (or fear of being reported as “impaired” by colleagues) may prevent a “cry for help.” Furthermore, loved ones often attribute behavioral changes to work-related stress.^21^ Many suicides result from acute events aggravating undiagnosed long-standing psychiatric illnesses, highlighting the need to systematically protect physicians from acting on transient suicidal impulses such as those relating to work or life events.^22^

#### Medicine judicialization and organizational oversight

Physicians are exposed to an array of stressors relating to judicialization of medicine and organizational oversight that go beyond the complexity of diseases and clinical cases.^23^ The interaction between the judicialization of medicine and organizations (e.g., federal/state levels, medical boards/oversight agencies, etc.) has a negative feedback loop on physician and patient health.^24-26^

Judicialization tends to raise costs and patient risk without improving health outcomes and leads to physicians dropping out of the system, sometimes due to suicide.^24^ In the UK, despite many physicians being under General Medical Council investigation (2010-2013), only 4.8% of the sharply rising complaints led to warnings/sanctions - but defensive medicine worsened.^27^ Defensive medicine raises US healthcare costs above all UK healthcare costs.^25^

The UK’s National Health Service reported that patient safety issues typically result from broad systems and conditions constraining healthcare staff.^28^ Malpractice risk increases with high clinical workloads, perhaps as there is little time to establish patient relationships and optimal clinical care.^26^ Malpractice risk also increases with age and male sex (both suicide risk factors).^29^ However, specialties at the highest risk of paid claims (mostly surgical specialties) do not have high suicide rates - and vice versa (psychiatry and anesthesiology pay less in claims but have higher suicide rates).^25,30^ Work ties strongly into physician self-worth^21^ and judicialization causes widespread stress as even settlements paid to cut insurer losses – irrespective of physician actions – are US National Practitioner Data Bank-reportable, leading to stigma and insurance/employment discrimination.^29^

Meanwhile, punitive organizational barriers relating to licensing, hospital privileges, and career advancement make physicians less likely to seek psychiatric help.^19,31,32^ Lower proportions of physicians than of the general public seek psychiatric treatment, partly due to fears about licensure and being unable to practice if investigated by oversight organizations. This has a compounding effect; e.g., suicides in physicians referred for fitness for duty evaluations (3.5%) were ~175 times higher than those in the general population.^33^ Nearly a quarter of deaths among physicians evaluated for fitness to practice by the General Medical Council (2005-2013) were due to suicide (n = 24) or suspected suicide (n = 4).^34^ Meanwhile, two-thirds of US state medical boards ask overly broad mental health questions on initial/renewal license applications, items that are not “consistent” with the recommendations of the American Medical Association, American Psychiatric Association, and Federation of State Medical Boards of the United States (FSMB) or with the American Disabilities Act. Physicians were less likely to seek help (OR 1.21) in those states,^19,31^ and most at-risk physicians are unaware of “safe harbor” provisions.^35^ Indeed, job-related problems are 3.12 times more likely to predict that suicides are in physicians vs. non-physicians; physician job satisfaction might be an intermediary in “a causal pathway between depression and suicide.”^7^

Medical organizations and their demands can also have major impacts on physicians’ romantic relationships and families. Our previous systematic review^3^ suggested that physician suicide risk increased not only with having relationship problems, being divorced or widowed (risks that may all increase during the pandemic), but also with being single (which may also be exacerbated by heavy workloads and living in smaller towns compounded by social distancing). The risk of divorce was roughly 1.5 times higher in female physicians compared to male physicians in a large survey of US healthcare professionals; furthermore, the divorce risk was higher in female physicians who worked over 40 hours/week compared to under 40 hours/week, while the opposite was seen for male physicians.^35^ This may imply a combination of social and organizational factors – e.g., how supportive a spouse may be of long work hours, stressors due to lack of organizational support for family leave/time off, particularly for women whose spouses/families expect them to bear the burden of child and homecare, and stressors to male physicians who want to work fewer hours or whose work hours may be limited by psychiatric comorbidities or other factors.

#### Burnout

Burnout affects 50% of physicians (being most prevalent in emergency medicine, which has been compared to a warzone),^36^ and is a risk factor for medical error and suicide.^37^

Under-recognition of physician needs and challenges by non-clinician administrators leads to systematic stressors, including excessive/misdirected regulations culminating in a “burnout crisis.”^36^ The NEJM Catalyst survey highlights the disconnect between healthcare executives and clinicians.^37^ It suggests that physicians tend to see systematic factors (including documentation/clerical work) they do not directly control as having the most significant impact on burnout. Yet most anti-burnout efforts target personal factors (e.g., physician resiliency), not core systemic problems. Burnout is driven by workload and inefficiency, lack of work autonomy and meaning, work-home conflict,^38^ negative patient relationships, and clerical tasks – especially those relating to electronic health records (EHRs).^39^ On reviewing the National Academy of Medicine Action Collaborative on Clinician Well-Being and Resilience Reference Collection, a recurrent association was found between burnout and lack of social connectedness^40^; a loss of belonging. Cornette and colleagues initially suggested the possibility of using the interpersonal psychological theory of suicidal behavior (IPTS) to understand physician suicide better,^41,42^ The IPTS components are perceived burdensomeness, thwarted belongingness, and acquired capability for suicide.^43^

Burnout was nearly twice as prevalent among physicians as among US workers in other fields after controlling for work hours and other factors.^44^ Between 2011 and 2014, the prevalence of burnout increased by 9 percent among physicians while remaining stable in other US workers.^44^ Several studies have also found a high prevalence of burnout and depression among medical students and residents, with rates higher than those of similar-aged individuals pursuing other careers.^44^ Cross-sectional studies have found burnout among physicians to be independently associated with 25% increased odds of alcohol abuse/dependence and 200% increased odds of suicidal ideation.^44^ In a longitudinal study of medical students, burnout predicted development of suicidal thoughts over the ensuing year, independent of symptoms of depression.^44^

Notably, while physician burnout is increasingly addressed in national and regional professional meetings, physician suicides are seldom brought up by professional associations, despite receiving some media attention.^20^

## Solutions

### Psychiatric comorbidities

The critical issue is early detection of psychiatric disorders and immediate and successful treatment of affected physicians. The Intern Health Study suggests that reaching out for help at the medical training level is more likely to prevent psychiatric conditions from being untreated and eventually affecting physicians’ practice and reputations.^45^ Consider the stress-vulnerability model, where vulnerability traits due to several factors (such as psychiatric comorbidities, history of suicide attempt, childhood-maltreatment) overlap with environmental stressors.^46,47^ If vulnerability traits are recognized early in training without stigmatizing trainees, and if this information is protected even from subpoenas, then vulnerable students and physicians can be given support and resiliency training early on and can maintain a support network throughout their careers. However, reducing adverse factors during training and practice that may cause negative mood symptoms (e.g., toxic work environments, lack of support, long work hours, sleep deprivation, etc.) may be even more important. Governments and non-governmental organizations should invest in online campaigns regarding consumption of alcohol and other “licit drugs”. Professional meetings and social events should avoid serving (especially free) alcohol or hard liquor. Making evidence-based online resources and interventions freely available at scale could benefit physicians’ mental health. Virtual meetings and similar resources can and should be used during the pandemic and similar future crises.^48^

Meanwhile, the WHO reports suicide reduction benefits following cognitive and problem-solving therapies, intensive care plus outreach, interpersonal psychotherapy, acceptance, and commitment therapy.^49^ Funding and supporting the development of these psychotherapies through telehealth might be an effective way to broaden suicide prevention during the pandemic and beyond. Many groups’ well-established “self-help” formats (Alcoholics Anonymous or Narcotics Anonymous) can be adapted to online formats while preserving anonymity. Widely implemented telepsychiatry or counselling maintaining privacy may be particularly appealing during the pandemic and beyond.^50^ Physicians may be more likely to seek out such services as they can avoid being seen in the psychiatrist’s office and possible career implications. Some initiatives like the Physician Support Line (+1 888 409-0141) (a free and confidential support line launched on March 30, 2020) are already connecting American physicians with volunteer psychiatrists.^51^

Mental health treatment may impact physicians’ ability to practice medicine, e.g., due to the cognitive side effects of medications or electroconvulsive therapy. Therefore, it is critical to manage psychiatric comorbidities at early stages, reducing the need for more extreme therapies. One possible option is non-invasive treatments such as repetitive transcranial magnetic stimulation, which is FDA-approved for major depressive disorder (and has early data on improving suicidal ideation and attempts),^52,53^ and transcranial direct current stimulation, which showed efficacy in multiple trials^54^ and was considered to be definitely effective (Level A) for depression.^55^ These techniques also show promise in substance use disorders.^56^ Such non-invasive brain stimulation techniques are relatively safe and typically have only minor transient side effects; physicians may thus be more likely to seek treatment if presented with these options, especially since protocols for improving working memory are similar to those used for some psychiatric conditions.^55^

Mandatory disclosure of psychiatric morbidities should be revisited by many of the State Medical Boards in the USA, thereby potentially reducing physicians’ self-treatment and allowing appropriate treatment.

### Judicialization of medicine and organizational oversight

Recommendations made after the General Medical Council investigation included reducing health examiner assessments, making physicians feel “innocent until proven guilty,” exposing investigational staff to frontline clinical practice, and establishing a National Support Service for physicians.^34^ The US FSMB adopted as policy the *Report and Recommendations of the Workgroup on Physician Wellness and Burnout*.^57^ However, there is a need for greater legal,^31^ legislative, and organizational advocacy led by physicians to protect physician rights and privacy, to enforce existing laws (e.g., ADA), and to create a culture of trust and transparency between healthcare workers, patients, and boards/oversight organizations.

It will be essential to reduce financial incentives for frivolous malpractice lawsuits (e.g., malpractice caps in Texas),^58^ provide obligatory standards for employer/insurer/medical board questionnaires and focus on measures that genuinely support physicians and healthcare organizations to improve patient care.

Physicians joining effective inter-professional health care teams and being encouraged to communicate openly may reduce stigmatization of psychological and work-related vulnerabilities, allowing doctors to ask for professional and personal help. Optimizing Suicide Prevention Programs and their Implementation (OSPI-Europe) contributed to reducing suicides in Europe; a similar program may help US suicide prevention.^59^

### Burnout

Training physicians may benefit from *compagnonnage* (an apprenticeship-based system of learning knowledge, skills, attitudes, and values within a context of mutual support),^60^ and adding “how to deal with future organizations” to formal trainee curricula. Workplaces should use effective strategies to reduce burnout, such as minimizing clerical tasks, redesigning practice,^36^ aligning physicians’/organizational values, increasing meaningful work,^61^ and, finally, by providing non-compulsory physician resiliency training.^37,62,63^ Furthermore, complementary strategies on burnout/suicide prevention for physicians should foster social connectedness.

In their 2019 article, Shanafelt and colleagues emphasize the need for all ethical healthcare organizations to address the issue of physician burnout, reinforcing the link between healthcare provider burnout, increased costs to the healthcare system, and patient harm including mortality, patient dissatisfaction, medical error, and workforce maintenance.^64^ They cite four main drivers motivating healthcare leaders to build sustainable and well-resourced well-being programs: (1) the moral-ethical case (provision of compassionate high-quality patient care); (2) the business case (overall costs to the healthcare system); (3) the tragic case (physician suicide); and (4) the regulatory case (lawsuits). They recommend measuring physician well-being longitudinally using an annual survey or scale, such as the Stanford model for healthcare provider well-being, based on the Professional Fulfillment Index which provides metrics for the healthcare organization and academic leadership on where to focus attention and resources.^65^ The metrics of success measured in this scale include three indicators of professional fulfillment: (organizational) culture of wellness, efficiency of practice, and personal resilience.

Additionally, a recently published call to action on physician burnout makes specific recommendations reinforcing many of the solutions suggested above^66^ and adds some more. It suggests “improved EHR standards with strong focus on usability and open APIs (Application Programming Interfaces).” Besides appointing chief wellness officers at the executive level, healthcare organizations should have engagement of key stakeholders (including health plans, insurers, the National Committee for Quality Assurance, state and federal agencies, medical school/residency programs, EHR vendors, healthcare organizations, and medical boards). Finally, it recommends proactive treatment and support for “burned-out” physicians (e.g., using statewide physician health programs (PHPs) such as Physician Health Services, Inc. in Massachusetts).^66^

## COVID-19 considerations

Effects of different countries’ COVID-19 responses on physician and population mental health are beginning to emerge. In China, frontline work was an independent risk factor for all-round worsened mental health; healthcare workers in Wuhan (and especially women and nurses) had more severe symptoms overall.^5^ Among young physicians in China, there were significantly worse daily mood symptoms, depression symptoms, anxiety, fear of violence, and observations of violence between Quarters 1 and 2 during the pandemic, unlike the previous year.^67^ Likewise, Italy had almost double the number of infected healthcare workers reported in China; with a large elderly population, Italian ICUs and healthcare facilities were under tremendous stress at the height of the pandemic.^68^

Physicians worldwide are often subject to complex/high-volume environments insufficiently supported for their workflow even under normal circumstances. During the COVID-19 pandemic, many physicians have been deployed (or volunteered) to work in unfamiliar care settings outside their realm of expertise (e.g., radiologists working on medical wards) and, in many cases, with insufficient personal protective equipment (PPE) and using PPEs beyond standard recommendations (e.g., reusing single-use masks).^69^ On top of all that, many suffer from the financial and moral injury of layoffs and salary cutbacks for physician and healthcare worker salaries,^70^ anti-lockdown protests, people refusing to wear masks, and personal attacks.^71^

This pandemic exacerbates the risk of burnout and moral injury. It adversely affects physician social support mechanisms with high workload and fears (for their own lives/health and those of their family, colleagues, and community). Despite easing of some restrictions during the pandemic, there is tremendous uncertainty as to physician risk of malpractice claims and punitive organizational actions when the pandemic is over, irrespective of physicians’ sacrifices for humanitarian reasons. It is expected that high work volumes, lack of adequate support, and unfamiliar work environments or clinical cases might increase the risk of provider error not only during the pandemic but also afterward, when backlogged elective cases might begin to flood the healthcare system.

The pandemic and lockdown may worsen suicide precipitants, e.g., domestic violence, feelings of social isolation, entrapment, loneliness, and grief,^50^ and alcohol/drug consumption,^72,73^ (especially with alcohol sales being treated as “essential services” and sales surging).^74^ Prolonged social distancing measures due to fear of transmitting the virus to their families exacerbates loss of belonging.^5^ An extra burden of stressors can substantially increase alcohol and substance misuse, relapse, and the potential for development of substance use disorders in at-risk individuals.^75^

Additionally, loss of jobs during the lockdown has highlighted the need for universal healthcare as opposed to employer-based plans in countries such as the US, and for better mental healthcare. Furthermore, easing licensing requirements across different states or regions while focusing on providing better care can reduce physician burdens and improve patient access to physicians.

The pandemic has also accelerated telemedicine/telehealth delivery and reimbursement, recovering some of physicians’ lost incomes and expanding telepsychiatry options for physicians in need. On the other hand, telemedicine best practices have not yet been consolidated and it will be essential to protect physicians working within reasonable parameters. If the flaws in the healthcare/financial/organizational structures are reproduced in the online environment, physicians may opt-out of medicine or turn to suicide in disillusionment and despair.

Medical students, trainees, and independent physicians have often demonstrated a strong and sincere desire to help in this pandemic; if adequately supported and if oversight mechanisms can be reorganized during this time of change to truly help physicians and their patients, we may see “posttraumatic growth” rather than stress disorders in physicians and the system as a whole.^76^

Finally, mental health consequences are likely to be present for more extended durations and peak later than the actual pandemic.^50^

### Limitations

Suicide reports vary by country and location for various reasons.^3,4,77^ One reason is that suicide data is collated from multiple incomplete and even overlapping sources.^3,4^ Different locations and organizations may face challenges in reporting or identifying suicides, may lack the resources to collect information, or data may not be collected systematically and reliably.^78^ These data may be incorrectly coded, whether due to lack of information availability (e.g., death of unclear intent), negligence, or intentional misrepresentation (e.g., physicians not reporting the death of a colleague as suicide to spare the feelings of the family and for life insurance), or omission (e.g., organizations not reporting suicides to avoid bad publicity). Hence, medical schools and healthcare organizations often do not fully record student and physician suicide deaths.^79^ The inconsistency and prominent subjectivity of coding race/ethnic data also leads to many challenges in different places. For example, in the US, Middle-Easterners from North Africa and Asia are typically classified as white, while those of mixed race might be classified as black/African American; Hispanics may be either white or black and may be coded separately, and Latino classifications are unclear. Furthermore, deaths of specific populations are often underreported, e.g., 33% of deaths of non-Hispanic American Indian or Alaska Natives are underreported.^80^

Multiple sources of information are useful to maximize validity within existing systems. For example, France’s Information System uses multiple databases to record, monitor and thereby reduce suicides: a medical-administrative database recording data on medical, surgical, and obstetrical admissions (PMSI-MCO) and psychiatric care (RIM-P), and the Oscour® network database that records post-suicide attempt admissions to Emergency Units.^81^ Regarding suicide attempts not reaching these systems, solutions include researching records of calls to emergency ambulance services to identify undocumented calls, noting that some requests will instead have been made to the Fire Brigade or to primary care doctors.

As to US physicians, considering the rather low likelihood of healthcare organizations reporting suicide deaths, gathering this information in more systematic ways is essential. Within the US Department of Labor, the Occupational Safety and Health Administration (OSHA) examines workplace fatalities to identify whether safety standards have been violated. Although the OSHA does not routinely assess suicides, many suicides occur in the workplace and can be considered workplace fatalities. Obliging healthcare facilities to publish anonymous aggregate data on physician suicides or inviting families to share this information could highlight this data to the OSHA. If OSHA evaluations systematically assessed suicides, the process would help improve workplace suicide estimates and identify risky or hostile working conditions for physicians and other staff in addition to patients.^82^

While the Centers for Disease Control and Prevention gathers annual data from hospitals on self-harm induced non-fatal injuries and collects survey data,^13^ it is crucial to improve our identification of suicide attempts as they are much more prevalent than death by suicide and are the most validated predictor of future suicide attempts.

Finally, the suicide-related consequences of the COVID-19 pandemic might vary depending on countries’ public health control measures, sociocultural, and demographic structures. For example, suicides by overdose (heavy drinking, or illicit or prescription drugs) in patients who may have been exposed to or who tested positive for SARS-CoV-2 may be underreported, as examiners may attribute death to COVID-19 infection rather than suicide. Conversely, in patients with known risk factors for suicide, deaths due to COVID-19 or accidental deaths due to alcohol/drug overdose or aspiration pneumonia may be misattributed to suicide, thereby over-reporting suicide deaths. Physicians may be particularly vulnerable as they are at higher risk of infection and of attempting to self-medicate the stress away. Furthermore, insufficient investigation into causes of death is likely in the context of high and/or rising death tolls during the pandemic, with mass graves being used to keep up in many countries and regions, e.g., Brazil,^83^ Bolivia,^84^ and the US.^85^

## Conclusion

Medicine used to be a prestigious and respected profession in which physicians exercised their autonomy both in healthcare approaches as well as in influence on public health authorities. This status is under threat as doctors’ mental health seems fragile and under severe pressure in the current system. Addressing systematic and organizational factors, followed by adequate healthcare for physicians, and, lastly, personal resilience training, should be the priorities for reducing modifiable factors contributing to physician suicide.

The pandemic has uncovered a significant capacity for change, and we should use this chance to heal the system and its most skilled workers: physicians.
